# Editorial: Diagnosis and treatment of bone metastases

**DOI:** 10.3389/fonc.2023.1247231

**Published:** 2023-08-15

**Authors:** Feifei Pu, Zuowei Hu, Yanping Yang, Ping Xia, Zhidao Xia

**Affiliations:** ^1^ Department of Orthopedics, Traditional Chinese and Western Medicine Hospital of Wuhan, Tongji Medical College, Huazhong University of Science and Technology, Wuhan, China; ^2^ Department of Orthopedics, Wuhan No.1 Hospital, Wuhan, China; ^3^ Department of Oncology, Traditional Chinese and Western Medicine Hospital of Wuhan, Wuhan, China; ^4^ Spine Institute, Shanghai University of Traditional Chinese Medicine, Shanghai, China; ^5^ Department of Orthopedics, Wuhan Fourth Hospital (Puai Hospital), Wuhan, China; ^6^ Institute of Life Science, Swansea University Medical School, Swansea, United Kingdom

**Keywords:** bone metastases, diagnosis, minimally invasive operation, radiotherapy, targeted therapy

Bone metastasis is one of the common complications of malignant tumors. Patients often present with bone pain and fracture, which not only seriously affect patients’ quality of life, but also imply poor prognosis (1, 2). In recent years, with the deepening of the understanding of the mechanism of bone metastases and the relevant regulatory network between tumor cells and bone microenvironment, the development and application of many new drugs have made great progress in the treatment of bone metastases (3). It is particularly important to find effective, economical, small adverse reactions and effective treatment methods to improve the quality of life of cancer patients. However, bone metastasis is often in the terminal stage of tumor development, the prognosis of patients is poor, and the treatment plan is difficult to be unified (4). Bone related biomarkers can reflect bone metabolism and bone turnover, and are associated with bone metastasis of malignant tumors (5). The development of diagnostic radiology is helpful to early identification of high-risk groups of pathological fractures, which is conducive to early intervention and improving the quality of life of patients (6). The incidence of bone metastasis is high, and an individualized comprehensive treatment plan should be developed according to the specific condition. The main treatment means include antitumor therapy, bone modifying drug therapy, surgery, radiation therapy, analgesia and supportive therapy (6–10).

In this Research Topic, 16 articles has been published which focusing on recent advances in the treatments for patients with bone metastases. Current published papers cover the following research areas: pathogenesis, establishment of animal models, pain management, imaging diagnosis, minimally invasive surgery and chemoradiotherapy of bone metastases.

Bone metastasis is a multi-step, continuous and extremely complex process involving both tumor and host factors. Yang W. et al. explained the epidemiology, clinical features, pathogenesis and clinical treatment strategies of bone metastasis in detail. They believed that with a better understanding of how bone metastases occur, there will be more new drugs and new technologies in the future, which will benefit more patients. Choosing the right animal model is an important bridge between basic research and applied research (11). Yu et al. summarized the current articles on the preparations and studies of animal models of bone metastases, including solid tumors such as lung cancer, breast cancer, and prostate cancer. This review is conducive to promoting the development of preclinical models and improving the translation of drugs and technologies for the treatment of bone metastases.

Early detection and treatment of bone metastases are of great significance to improve the prognosis of patients. Yang et al. reviewed the biomarkers related to bone metastasis, hoping to provide guidance for the early diagnosis of bone metastasis. Biomarkers can effectively reflect the occurrence, progression, tumor treatment monitoring, recurrence detection and so on. Brouns et al. found that the expression of nuclear factor κB ligand receptor activator (RANKL) gene and the increased ratio of RANKL: osteoprotegerin (OPG) were associated with the presence of bone metastases. The study also showed that an increased proportion of the RANKL: OPG gene was associated with a higher incidence of bone metastasis. The study suggested that research into the mechanism of bone metastases may facilitate the development of new drugs and may change the entire treatment strategy.

In recent years, deep learning based on big data is developing rapidly, and the biological behavior of tumors can be reflected by the texture features of lesion areas that are difficult to be recognized by the naked eye through imaging omics (12). The algorithm extracts abstract features of tumor regions through multi-layer network structure (13). Long et al. developed an ensemble machine-learning model for predicting early mortality among patients with bone metastases of hepatocellular carcinoma, Huo et al. developed a deep learning-based algorithm in lung cancer bone metastases detection on computed tomography, and Cui et al. developed a web-based calculator to predict three-month mortality among patients with bone metastases from cancer of unknown primary. Surveillance, Epidemiology, and End Results (SEER) database provides good data support for clinical research, Nomogram has been widely used in cancer and other medical research because of its intuitive and convenient characteristics (14). Yin et al. constructed a novel web-based nomogram for lung cancer patients with bone metastasis, they found the prediction models may be helpful for doctors to make accurate judgment and guidance on the treatment plan and clinical prognosis of patients.

Bone pain is the main clinical symptom of patients with bone metastatic cancer, and also the main reason for the decline of patients’ quality of life. Therefore, lasting and effective pain relief is the focus of clinical treatment. Jing et al. reviewed the management of pain in patients with bone metastases, which has important guiding significance for the majority of clinicians. Denosumab (the RANKL neutralizing antibody) can not only relieve pain symptoms in patients with bone metastases, but also prevent the occurrence of bone-related adverse events (15). Lu et al. reviewed current comprehensive understanding the pharmacological action and clinical trial results of denosumab in the treatment of bone metastases.

At present, clinicians are increasingly recognizing the importance and scientific nature of multiple disciplinary team (MDT) in the diagnosis and treatment of bone metastases. As an important means of treatment, surgery can be divided into different surgical methods according to different therapeutic purposes. The surgical goals of bone metastases in the extremities are to prevent and treat pathological fractures and to control the tumor locally (16). Pu et al. found that total removal of bone metastases and implantation of personalized modular prostheses can reduce pain and improve limb function and quality of life in patients with femoral shaft metastasis. Wu et al. preserved rectus femoris after total femoral prosthesis replacement following resection of femoral malignant tumors, this could improve limb function.

Treatment strategies for metastatic tumors of the extremities and spine differ. Surgical goals for metastatic tumors of the spine are local tumor control, pain relief, spinal stability, relief of spinal cord neurologic compression, and improved quality of life (16). Positive vitreous pressure (PVP) and percutaneous kyphoplasty (PKP) can effectively relieve the pain degree of spinal metastasis patients, with the advantages of small trauma, low risk, short operation time and quick analgesic effect (17). Wang et al. Pulmonary cement embolism is a rare, Wang et al. investigated risk factors for pulmonary cement embolism after PVP and radiofrequency ablation for spinal metastases. But underestimated complication of vertebroplasty, Zhang C. et al. specifieded the direct and indirect damage zone of radiofrequency ablation in porcine lumbar vertebra by conducting animal experiments, this will improve the guidance for improving the safety of the operation. According to literature reports, about 60% of spinal metastases are tumors with abundant blood flow. Arterial embolization can not only be used as an auxiliary means before surgery to reduce hemorrhage in the hand and improve the safety of surgical operation, but also as palliative treatment. Vascular embolization can cause tumor ischemic necrosis and relieve patients’ pain and tumor compression and other symptoms (18). Zhang B. et al. found that preoperative embolization is an effective and safe method to control bleeding in patients with metastatic epidural spinal cord compression. Spinal metastasis of malignant adrenal tumor (SMMAT) are rare malignant neoplasms originating from the adrenal glands. Liu et al. reported six cases of SMMAT, and they elucidated the clinical characteristics and discussed surgical management and outcomes of SMMAT.

At present, the treatment concept and technology of bone metastases have developed rapidly, and there are still many clinical problems to be solved. After the occurrence of bone metastasis in a certain pathological type of cancer, the specific selection of radiotherapy, surgery, systemic therapy, targeted drugs, immunotherapy, its sequence and the details of the basis of combination are one of the important clinical issues at present, which still need clinical research and practice verification. According to the practical experience of MDT for cancer bone metastasis, it is one of the important directions of clinical research to evaluate the classification of different cancers and select treatment strategies in the future.

## Author contributions

All authors listed have made a substantial, direct and intellectual contribution to the work, and approved it for publication.
